# Urgent call for action: avoiding spread and re-urbanisation of yellow fever in Brazil

**DOI:** 10.1590/0074-02760170361

**Published:** 2017-11-27

**Authors:** Cristina Possas, Reinaldo Menezes Martins, Ricardo Lourenço de Oliveira, Akira Homma

Yellow fever (YF), a haemorrhagic viral disease with a high fatality rate, is considered one of the greatest scourges of mankind. Historically, devastating epidemics of this disease have been reported since the seventeenth century in Africa, the Americas, and Europe ( WHO 2017 ).

The control of urban YF in Rio de Janeiro in the beginning of the last century is recognised as one of the most successful public health initiatives in the world. Oswaldo Cruz’s successful campaigns with a strong and enforceable fight against the domestic mosquito vector *Aedes aegypti* dramatically dropped the number of urban YF cases in the city. The measures were applied with success across the country. The elimination of urban transmission in Brazil and all over the Americas was achieved in the early 1940’s with a combination of vaccine campaigns and *Ae. aegypti* eradication ( Franco 1969 ). However, the ineradicable zoonotic sylvatic transmission cycle maintained between *Haemagogus* and *Sabethes* mosquitoes and nonhuman primates (NHPs) has annually caused infections and death in humans. Importantly, YF virus (YFV) epizootics have cyclically been reported every 7-14 years in Brazil, with marked special expansion in the past decades. The last two epidemics in Brazil spanned more than one year: from 1998 to 2003, and again in 2008 and 2009, despite vaccine campaigns ( Vasconcelos 2010 , Jentes et al. 2011 , MS/SVS 2017 ).

The recent sylvatic YF outbreak in Brazil has been the most severe in the last seven decades and became an epidemic. As of 31 May, 2017, a total of 3240 cases have been reported in Brazil: 792 confirmed, 1929 dismissed, and 519 remain under investigation, including 435 deaths (274 confirmed, 124 discarded, and 37 under investigation). The case fatality rate (CFR) among confirmed cases is 34.5% ( MS/SVS 2017 ). For NHPs, 3850 epizootics were reported (642 confirmed, 96 dismissed, and 1448 under investigation).

The rapid spread of YF cases in Brazil has led to a major concern: infections were no longer reported just in the jungle and remote inland rural areas, but sylvatic transmission also occurred in the surroundings of the most densely populated cities in the states of São Paulo (SP), Minas Gerais (MG), Espírito Santo (ES), Bahia (BA), and Rio de Janeiro (RJ) ( MS/SVS 2017 ). It started in MG late in 2016. Despite being an area where routine immunisation is recommended and there is good vaccination coverage in the young population, the epidemic reached and hit with severity the rural area of north-eastern MG, due to poor vaccine coverage among adults. The rapid spread to the coastal state of ES, an area where YF vaccine was not routinely recommended, led to a severe epidemic with 260 confirmed cases and 85 deaths. Subsequently, the virus also spread to RJ, which also had no previous recommendation for routine YF vaccination. Fortunately, some of the areas at higher risk in RJ had already been partially vaccinated at this time. Nevertheless, 17 confirmed cases and seven deaths were recorded in the state from March to June 2017 ( MS/SVS 2017 ).

The YFV circulating in Brazil since 2008 belongs to the South American Genotype I, lineage 1E ( Cardoso et al. 2010 , Souza et al. 2010 ). The whole genome of YFV causing the 2017 outbreak in ES was sequenced and phylogenetic analysis revealed that it clusters in the 1E sub-clade along with recent Brazilian and Venezuelan strains ( Mir et al. 2017 ). However, it was discovered that this virus strain displays seven new mutations, and their role in host infectivity and virus replication and spreading is still under investigation ( Bonaldo et al. 2017 ). The unique amino acid signatures carried by the 2017 YFV occur in non-structural proteins, one being a change in the capsid protein and the other in components of the viral replicase complex, the NS3 (two changes), and the NS5 (five changes) proteins; no change was detected in the envelop protein. Although the amino acid changes may affect the viral fitness, they would not potentially impact the efficacy of the available vaccines ( Bonaldo et al. 2017 ).

A complex combination of ecological, social, and behavioural factors may help to explain the severity and efficient spread of the YFV in Southeast Brazil, particularly its dissemination to the Atlantic coast. Among them, ecological issues included changing environmental and climate conditions favouring a high density of competent sylvatic and urban vectors and primary amplifier vertebrate hosts (NHPs); the uncontrolled occupation of forest areas by human populations; and the large number of susceptible NHP and nonvaccinated people in peri-urban areas in close contact with forests, mainly the adult human populations from rural areas in north-eastern MG affected by the recent YFV outbreak as well as in vast coastal receptive zones ( Vasconcelos 2010 , Couto-Lima et al. 2017 ). In these zones, lack of access of populations to sanitation and garbage collection in urban areas, favouring *Aedes* proliferation, is contributing to an increase in the potential for re-urbanisation of YF in Brazil.

YF epidemics are not acceptable and are an ethical issue. Brazil is the largest producer of the YF vaccine, which is provided free of charge. Vaccination was not recommended universally in Brazil due to the serious adverse events associated with it, 0.42 cases/100,000 administered doses in Brazil, similar to those found in Africa. In the United States, Europe, and Australia, reported rates of serious adverse events are about ten times higher. The reasons for such large differences are not clear, but intensity and quality of surveillance are crucial ( Staples et al. 2008 , Martins et al. 2010 , 2014 , MS 2014 ). Now the risk of transmission has increased, and the risk-benefit balance favours universal vaccination. This action is urgent, taking advantage of the temporary relief in YFV transmission in most of Brazil caused by the reduced rainfall and lower mean temperatures recorded during the dry season (July-October). These environmental conditions reduce the availability of mosquito larval sites, larval developmental time, mosquito adult longevity, and viral replication. YFV epizootics and human cases will potentially re-emerge in the following rainy season. The last two epidemics in Brazil spanned more than one year: from 1998 to 2003, and again in 2008 and 2009, despite vaccine campaigns ( Vasconcelos 2010 , Jentes et al. 2011 , MS/SVS 2017 ).

In this aftermath, the decision by the Ministry of Health to vaccinate all the Brazilian population against YFV was obvious, and received strong support from the Brazilian scientific community and international health authorities. Due to the sudden increase in demand and limited vaccine supply, the decision by the Ministry of Health to administer only one dose of the vaccine, as recommended by the World Health Organization, was provisionally endorsable. Nevertheless, booster doses are necessary to ensure longer protection for all YFV vaccines ( CGSYF 2014 , Campi-Azevedo et al. 2016 ). Therefore, the emergency decision to administer only one dose of the vaccine recently taken by the Brazilian Ministry of Health should be re-evaluated as soon as the current sanitary situation normalises and new information on duration of immunity is available.

YF vaccination needs to be urgently intensified before the next rainy season, chiefly in the states with the highest risk, such as RJ and other coastal receptive and vulnerable Brazilian zones. RJ has the largest urban forest in the world. Large remains of the Atlantic rainy forest extend in a vast territory from South to Northeast Brazil, along which millions of susceptible people live in cities, villages, and farms under the influence of this biome. Competent sylvatic mosquito vectors and susceptible NHPs are also largely distributed in this area. All together, these features render this large territory very receptive to sylvatic YFV re-emergence in the next rainy season. Alarmingly, this territory also reports high infestation by *Ae. aegypti* , and also of experimentally YFV-susceptible *Ae. albopictus* populations *.* The proximity with the YFV sylvatic transmission foci and the intense people movement in this territory combined with high density of these urban vectors increase the risk of YFV re-emergence in an urban cycle, which needs to be urgently prevented with vector control measures and vaccination campaigns.

YF vaccination needs to be implemented for the entire Brazilian population, with specific recommendations for groups with higher risk of serious adverse events. Mobile immunisation units should be urgently provided to reach the more vulnerable rural populations who have limited access to immunisations in health centres.

In addition, as the demand for YF vaccine will continue to increase due to the epidemiological context in Brazil and elsewhere, there is an urgent need to strengthen public manufacturer’s capacity to provide the YF vaccine to all who need it at an affordable price.

Cristina Possas  Reinaldo Menezes Martins  Ricardo Lourenço de Oliveira  Akira Homma
